# Efficacy and Safety of Cladribine: Subcutaneous versus Intravenous Administration in Hairy Cell Leukemia Patients

**DOI:** 10.4084/MJHID.2015.058

**Published:** 2015-10-16

**Authors:** Ola Khorshid, Alfred Elias Namour, Mosaad M El-Gammal, Tarek Yakout Mahmoud, Catherine Fortpied, Raafat Abdel-Malek, Safaa Ramadan

**Affiliations:** 1 National Cancer Institute, Cairo University, Cairo, Egypt; 2 Kasr Al-Ainy school of Medicine, Cairo University

## Abstract

Cladribine induces durable complete remission (CR) in approximately 85% of hairy cell leukemia (HCL) patients. In Egypt, cladribine is mainly used as IV continuous infusion at a dose of 0.1 mg/kg/day for 7 days and as SC bolus injection at a dose of 0.14 mg/kg/day for 5 days. We aimed to compare the outcome and toxicity between these two regimens. We retrospectively collected data from HCL patients treated at the National Cancer Institute and its affiliated center, Nasser Institute, Cairo, Egypt. Forty-nine patients were identified, 18 treated with the IV regimen (IV group) and 31 with the SC regimen (SC group). Forty-one patients were newly diagnosed. Patient characteristics were balanced across the two groups. The CR rates in the IV and the SC group were 94% and 97%, respectively. The main complications in the IV group and the SC were neutropenia G3–4 (67% vs. 87%), mucositis mainly G1–2 (67% vs 32%) and infections (mainly viral, 78% vs 34%). In the IV group, five patients died, three of progression and infection, one of unknown cause and one of late heart failure. In the SC group, one patient died of disease progression and one of second cancer. After 33.5 months, median follow-up, the 3-year event free survival was 60% and 96%, respectively (p=0.104). The 3-year overall survival was 81% and 100%, respectively (p=0.277). In conclusion, SC cladribine is an excellent alternative to the IV regimen for the treatment of HCL.

## Introduction

Hairy cell leukemia (HCL) is a rare indolent B-cell leukemia. It accounts for approximately 2% of all leukemias and is characterized by male preponderance (M:F ratio is 4:1). The majority of patients are usually diagnosed over the age of 40 years. Main symptoms are related to cytopenia and/or splenomegaly.^1^,^2^ The diagnosis is confirmed by bone marrow evaluation with flow cytometry. A panel of pan-B cell markers and antigens commonly expressed on hairy cells (CD19, CD20, CD22, CD103, CD11c, CD123 and CD25) is used to diagnose HCL cells.^1^–^4^ Annexin A1, which is not expressed in any other small B-cell lymphoproliferation, is currently the most specific HCL marker.^1^,^5^ Tiacci et al have identified BRAF-V600E mutation as the disease-defining genetic event among patients with classical HCL.^6^,^7^

Indications for treatment are the presence of constitutional symptoms, symptoms due to splenomegaly, significant cytopenia (absolute neutrophil count (ANC) <1×10^9^/L, platelet count < 100 × 10^9^/L, or hemoglobin level <10g/dL), recurrent infections and autoimmune complications. Main treatment objectives are to control symptoms, normalize blood counts, and achieve lengthy remission. Over the years treatment options have evolved from moderate success with low-dose chlorambucil, splenic irradiation or even anthracyclines, through splenectomy and IFN-α, and eventually to the current treatment of choice: the purine nucleoside analogues pentostatin and Cladribine.^1^,^2^

Excellent outcomes are obtained with purine analogues and true primary resistance is seldom observed.^1^,^2^,^8^,^9^ CR can be achieved in 80–85% of patients and more than 90% of patients are alive at 10 years.^10^,^11^ Relapse rate is about 40% in the first 5–10 years and is rare after 10 years.^1^,^2^,^10^–^15^ Second CRs can be obtained in more than 75% of patients using the same agents.^12^,^15^

Cladribine can be administered via intravenous (IV) or subcutaneous (SC) route. The 7-day IV continuous infusion of cladribine at a dose of 0.1 mg/kg/day is considered the standard regimen in the treatment of HCL patients.^1^,^2^,^16^–^20^ Alternative treatment schedules such as short daily or weekly infusion.^8^ The SC injection produces equal (100%) cladribine bioavailability as IV.^21^,^22^ Cladribine is the first treatment choice due to its convenient administration schedules that seems to be more advantageous to both patients and physicians.^1^,^2^

At the NCI- Cairo, Egypt, the two commonly used regimens of cladrabine are IV continuous infusion at a dose of 0.1 mg/kg/day for 7 days^10^ and SC bolus injection at a dose of 0.14 mg/kg/day for 5 days.^23^ In this study, we compared outcome and toxicity among HCL patients treated with cladribine by SC bolus injection to patients treated with clardribine by continuous IV infusion.

## Patients and Methods

### Patients

We retrospectively collected data of HCL patients treated with clardribine during the period 2003 and 2010 at the National Cancer Institute (NCI)-Cairo and Nasser Institute, an NCI- Cairo affiliated center. Eligible patients could have been treated with single or two courses of cladribine. The study was approved by the Ethics Committee of the NCI- Cairo. The diagnosis of HCL was done based on hematopathology review of the peripheral blood, bone marrow, and/or splenic tissue in splenectomized patients as well as the characteristic immunology profile of hairy cells in the bone marrow or the peripheral blood.^3^,^4^ The analysis of BRAF mutations was not done to any of the patients as it is not part of our standard of care. Patients should have received clardribine either by IV (IV group) or SC route (SC group) as previously described.^10^,^23^

Medical records were reviewed for date of diagnosis, gender, age, performance status, initial complete blood count, bone marrow aspirate and biopsy results, organ involvement, immunophenotyping, prior therapies, route of cladribine administration, treatment outcomes and toxicities, last date of follow-up, disease and patient status at last follow-up, date of disease progression, and date and cause of death.

### Treatment response and toxicity

Cladribine was given at a dose of 0.1 mg/kg/day for 7-days by continuous IV infusion to 18 patients and at a dose of 0.14 mg/kg/day for 5-days by SC injection to 31 patients. The use of either the IV or the SC route was approved during the period of the study and patients in each group were treated concurrently. The choice of the regimen was according to the discretion of the physician.

Complete and partial remissions are assessed when patients are recovered from cytopenia (usually between month 3–6 post-treatment). Responders (CR or PR) and non-responders are defined according to the Consensus Resolution.^1^,^24^,^25^ In our experience, clearance of hairy cells from the bone marrow might take 4–6 months. The same length of time is sometimes required to document normalization of the size of involved spleen, liver and lymph nodes. So, evaluation of response between the 4th and 6th months of treatment is critical to decide if a second treatment course is needed, in particular if massive organomegaly or severe cytopenia still exists.^25^ Patients are then followed every three months for the first year and then annually by peripheral blood counts only.

The median and range of blood values at day 10 (nadir) were compared between the two groups. Hematologic and non-hematologic toxicities are graded according to the Common Terminology Criteria for Adverse Events (CTCAE) Version 4.0.

### Statistical methods

Overall survival (OS) was defined as the time elapsed between the start of cladribine treatment and death or loss to follow up. Event free survival (EFS) was defined as the time elapsed between the start of cladribine treatment and progression, relapse, second tumor, or death, whichever occurred first. Time to event endpoints was analyzed using Kaplan-Meier method. Comparison between the two groups (subcutaneous versus intravenous route) was performed using log-rank test for survival endpoints, Student t test for continuous variables and Fisher’s exact test for categorical variables.

A univariate analysis was conducted in order to assess the prognostic value of the following baseline characteristics on EFS: age (>= 50 versus <50 years), gender, lymphadenopathy and/or hepatomegaly (yes versus no), previous treatment (nil, chemotherapy, Interferon, splenectomy,) and route (SC versus IV). The prognostic value of response to treatment at 4 months on EFS (complete versus partial response) was evaluated using landmark analysis with a landmark time of 4 months. These prognostic factor analyses were performed using a Cox proportional hazards model and Wald test was used to assess the statistical significance of the factors. However, the low number of events for EFS should be considered in the interpretation of the results.

All tests were performed at the 5% two-sided significance level. The statistical analysis was conducted using SAS® version 9.4.

## Results

### Clinical characteristics of patients

The relevant characteristics of patients in each treatment group are compared in Table 1. A total of 49 patients were identified: 18 patients in the IV group and 31 in the SC group. Forty-one patients were newly diagnosed and 8 were previously treated. Previous treatment was chemotherapy (n=4), interferon alpha (n=2) and splenectomy (n=2). The median age was 47, range 26–74 years and 78% were males. The clinical characteristics were overall well balanced for patients across the IV and SC groups.

### Responses to cladribine

After treatment with cladribine, CR was achieved in 94% of patients (17/18) in IV group and in 97% of patients (30/31) in the SC group (Table 2A). At first evaluation post-treatment (months 3–4), 12 patients were still in PR: two in the IV and 10 in the SC group. At the subsequent evaluation, in the IV group one patient achieved CR and the other died of disease progression and infection neutropenia. Whereas, in the SC group, 9 patients could reach CR, 5 with no further therapy and 4 following a 2nd cycle of clardribine. The tenth patient progressed after the 2nd cycle of clardribine and was lost to follow-up. This patient was previously treated with combination chemotherapy and presented with splenomegaly and lymphadenopathy.

We also did the same analysis restricted to the subset of patients who received cladribine at onset of disease and with single cladribine course in the IV and SC group. CR rates were not different between the two groups, but again time to CR was longer in the SC group. CR rates at week 12 and at subsequent evaluation were 64% and 100% in the SC group, while it was 93% at the two time points in the IV group (Table 2B).

### Toxicities

Blood values at diagnosis and at day 10 (nadir) and non-hematologic complications are presented in Table 3A and B. At day 10, there was no difference in the changes in the median blood values between the two groups. Neutropenia G3–4 in the IV group and the SC occurred in 67% (n=12) and 87% (n=27) of patients, respectively. Mucositis was significantly more in the IV group compared to the SC group: 67% (n=12) vs 32% (n=10) P <0.001, respectively. Three patients in the IV group had G3 mucositis and all other patients had GI-II mucositis.

As regard to fever and infectious complications, the rates were 78% (n=14) and 34% (n=11), respectively. Infections occurred mainly within 10 weeks after treatment. In most patients, the infectious agent couldn’t be identified. However, herpes simplex infection was documented in 6 patients (5 in the IV group and 1 and the SC group). In each group, one patient suffered from fungal infection. Fever without any identified source of infection was observed in 4 patients in the IV and in 7 in the SC group.

In the SC group and following the 2nd cycle of treatment one patient had mild bilateral foot drop and one had mild shoulder tendinitis.

Other non-hematologic toxicities were mild (G1–2) and in the form of nausea, vomiting, elevated liver enzymes, cough, headache and bony pains.

Late toxicities included heart failure in one patient in the IV group and second cancer in one patient in the SC group. Causes of death in the IV and SC groups are summarized in Table 3C and are detailed below.

### Event Free Survival

**Figure 1:**
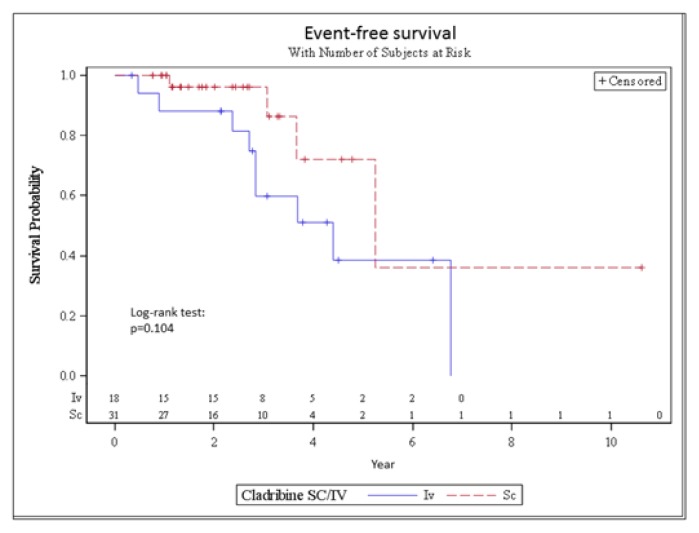
EFS of the IV and SC cladribine group.

The median time to follow up was 33.5 months (range, 10–28 months). Median EFS were 53 and 63 months in the IV and the SC group, respectively. The 3-year EFS was 60% and 96% in the IV and the SC group, respectively (p=0.104) (Figure 1).

In addition to the patient died shortly after progression, another patient died of unknown cause while in CR at month 11 post-therapy. Furthermore, seven patients relapsed in the IV group. Of them, two died shortly after progression due to infectious complications and five could obtain second remission after SC cladribine. However, one of them died in CR after 2 years of follow up due to heart failure. This patient presented with positive history of ischemic heart disease.

In the SC group, one patient died of disease progression and two experienced relapsed at 44, 13 and 37 months, respectively. Both relapsed patients were re-treated with SC clardribine, one achieved PR and was lost to follow–up, and the second obtained CR. Additional patient was diagnosed with diffuse large B cell lymphoma 70 months from the initiation of therapy. This patient died during treatment due to tumor lysis syndrome.

**Figure 2:**
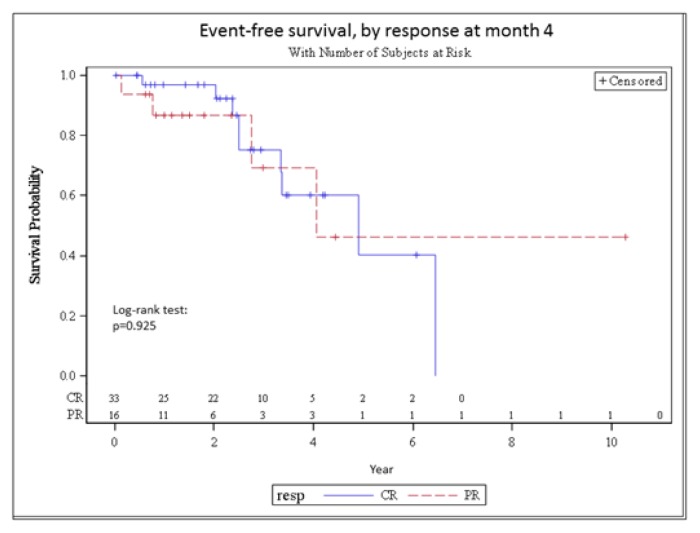
Event Free Survival, by response at month 4 (CR versus PR) – Landmark analysis. Months counted from the landmark time of 4 months.

Clinically relevant prognostic factors that would influence the EFS were analyzed and the results are shown in table 4. Results suggests that having lymphadenopathy and/or hepatomegaly is of poor prognosis (HR=6.7, 95% CI= 1.3–33.9, p=0.023). No other factors, including route of administration, showed a significant prognostic effect in this analysis. The analysis of EFS by response to treatment did not show any difference between patients in CR and patients in PR at months 4 (p=0.925) (Figure 2).

### Overall survival

**Figure 3:**
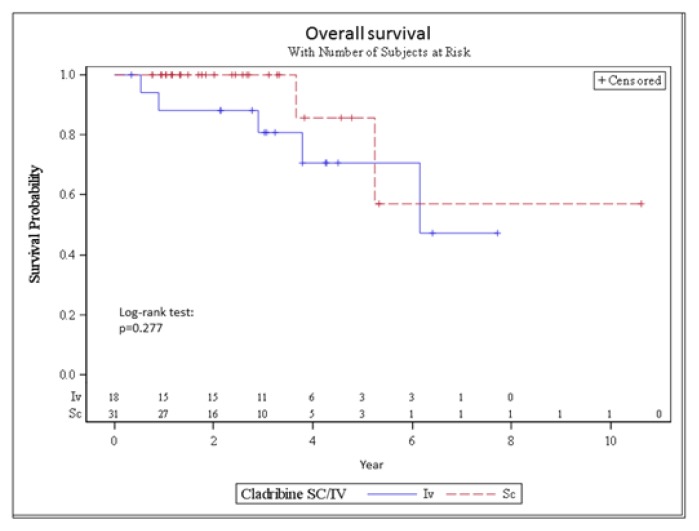
OS of the IV and the SC group.

Median OS was 74 months in the IV group and was not reached in the SC group. The 3-year OS was 81% and 100% for patients in the IV and SC group, respectively (p=0.277) (Figure 3).

## Discussion

To our knowledge, this study that compared HCL patients treated with SC cladribine vs IV cladribine is the first from a developing country. We reported the clinical features and the outcome of 49 Egyptian HCL patients treated with cladribine in the period between 2004 and 2010. The clinical presentation of patients in this series is similar to the literature.^1^ The results show that SC cladribine is safe and produces a remission rate similar to the IV route. Importantly, the two regimens resulted in high EFS. The SC route was associated with non-significant but important longer DFS (median 63 vs 53 months and 3-year EFS 96% vs 60%). EFS than the IV regimen.

The overall CR rate of this series was 96% which matches well to CR rates reported in previous studies which ranged between 87%–100%.^1^,^2^ Interestingly, the time to achieve CR was longer in the SC group (table 2A and B), and 5 patients required a second course of cladribine to reach CR. While 89% of patients were in CR at first evaluation in the IV group, only 68% of the SC group reached CR. At the subsequent assessment the rates improved to 94% and 97%, respectively. Studies investigating the efficacy of the 7 day and 5 day regimens of SC cladribine reported CR rates of 81% and 76%, respectively.^23^,^26^ Therefore, in this study, the estimated CR rate is very encouraging for SC cladribine.

Cladribine is a generally well-tolerated^1^,^2^,^23^,^26^–^29^ drug, and cytopenia and culture negative fever are the most commonly expected side effects.^1^,^2^,^27^ Infections are also a common toxicity and occur mainly during the first weeks of treatment along with cytopenia.^1^,^2^,^27^ In a review by Maevis et al, the frequency of fever among HCL patients treated with cladribine ranged between 40–69%, and not all fevers were associated with signs of infection.^2^ The authors suggested that fever may be a sign of cytokine release in HC rather than infection in some patients.^2^ In accordance, the most frequent toxicity in this study was a myelosuppression (approximately 80% of patients). Fever (with or without signs of infection) was seen in 35% of all patients, and infections were diagnosed mainly in the first 10 weeks post-therapy. Nevertheless, infectious episodes (mainly herpes simplex) and mucositis were more frequent in the IV group.

A number of investigators have indicated that the risk of developing second malignancy is higher in HCL patients.^18^,^30^ Whether this is related to common genetic susceptibility or due to treatment exposure is not known. In a study of 358 patients with HCL treated with cladribine, 8% (27/358) developed second tumors.^18^ According to the Surveillance, Epidemiology and End Results (SEER) data, of 3104 patients with HCL, the incidence of second cancer was 32% compared to 23% in the general population (SIR=1.2; 95% CI 1.1–1.4 for all, 6.6 for Hodgkin lymphoma, 5.0 for non-Hodgkin lymphoma and 3.6 for thyroid cancer.^30^ In our study, one patient (2%) died of diffuse large B cell lymphoma 70 months after CDA therapy. Without longer follow up, analysis of late effects such as second cancers will not be valuable.

In a recent literature review, the 5-year PFS of patients treated with cladribine was found to be in the range of 72–84%.^2^ In correspondence to this range, the overall 3-year EFS of patients in this report was 77%. However, patients in the IV group experienced more failures than patients in the SC group (3-year EFS was 60% vs 96%) and median EFS was shorter in patients in the IV than in the SC group (53 vs 63 months, respectively). This trend did not reach statistical significance in the univariate analysis. A number of studied evaluated SC cladribine as alternative to the IV route. In one study no relapse was seen after 20 months follow up of 73 HCL patients treated with the 7 day SC schedule.^26^ In the second, 62 patients received the 5 day SC regimen and the 18 months-EFS was 68%.^23^ A comparison between the different results is not possible due to variability of end point definitions and methods on analysis.

Different prognostic factors have been previously described, such as cytopenia^1^,^12^,^13^ and lymphadenopathy at presentation^14^,^31^ and response to purine analogues.^12^,^14^,^25^,^29^ Two main studies indicated response to purine analogs as the most significant factor for DFS.^12^,^25^ Therefore we compared EFS between patients in CR and patients in PR and no statistically significant difference between the two subgroups was found.

Albeit non-significant, the 3-year OS for patients treated with the SC regimen was 100% compared to 81% in patients treated with the IV regimen. This is very supportive to the effectiveness of the SC regimen. Main causes of death in the IV group were complications related to either disease progression or treatment re-challenge.

Being a rare condition, conducting prospective studies in HCL is a real challenge. Limitations to retrospective studies include heterogeneity in data recording and data collection as well as variability in treatment decisions among centers. However, data collected from single center are usually more homogenous as is the case of the current series. Therefore, this study would provide valuable information on such rare disease.

In conclusion, this is the first report of a large series of Egyptian HCL patients comparing the SC to the IV cladribine regimen. Our data confirms that SC cladribine is an excellent alternative to the IV regimen. SC cladribine is well tolerated and is associated with favorable EFS. Together with the convenience of the SC route, this regimen should be recommended as of choice to treat HCL patients in institutes with limited resources such as those in developing countries.

## Figures and Tables

**Table 1:** Baseline characteristics: overall and by treatment group (IV versus SC).

		All patients (N=49)	IV (N=18)	SC (N=31)	P value (*)
Sex	Male	38	15	23	0.724
	Female	11	3	8	
Age	Mean ± SD	47.1±9.9	46.5±8.0	47.5±11.0	0.733
	Median (range)	47 (26–74)	47.5 (33–65)	46 (26–74)	
Status of study entry	1^st^ line	41	15	26	1.000
	1^st^ relapse	8	3	5	
Splenomegaly	Present	46	16	30	0.546
	Absent	3	2	1	
Lymphadenopathy	Present	9	4	5	0.708
	Absent	40	14	26	
Hepatomegaly	Present	3	0	3	0.288
	Absent	46	18	28	
Performance status	0	42	13	29	0.084
	1	7	5	2	
Previous therapies	Chemotherapy	4	0	4	0.092
	Interferon	2	1	1	
	Splenectomy	2	2	0	
	Nil	41	15	26	
Hemoglobin (g/dl)	Median (range)	8.4 (4.4–10.9)	8.8 (5.3–10.6)	8.2 (4.4–10.9)	0.272
Leukocytes (× 10^9^/L)	Median (range)	2.6 (0.8–26.2)	2.95 (0.8–26.2)	2.4 (0.9–23.0)	0.499
Platelets (× 10^9^/L)	Median (range)	38 (3–206)	39 (16–98)	33 (3–206)	0.555

(*) For the comparison between the IV and SC groups (Fisher’s exact test or t-test).

**Table 2A:** Response: overall and by treatment group (IV versus SC).

Time point	Response	All patients (N=49)	IV (N=18)	SC (N=31)	P value (*)
4 weeks	CR	7 (14%)	3 (17%)	4 (13%)	0.697
	PR	42 (86%)	15 (83%)	27 (87%)	
8 weeks	CR	25 (51%)	12 (67%)	13 (42%)	0.140
	PR	24 (49%)	6 (33%)	18 (58%)	
12 weeks	CR	37 (76%)	16 (89%)	21 (68%)	0.168
	PR	12 (24%)	2 (11%)	10 (32%)	
At subsequent assessment of response	CR	47 (96%)	17 (94%)	30 (97%)	1.000
	Relapse/progression	2 (4%)	1 (6%)	1 (3%)	

(*) For the comparison between the IV and SC groups (Fisher’s exact test).

**Table 2B:** Response in subset of patients treated at onset of disease and with a single cladribine course: overall subset and by treatment group (IV versus SC).

Time point	Response	Overall subset (N=37)	IV (N=15)	SC (N=22)	P value (*)
4 weeks	CR	4 (11%)	2 (13%)	2 (9%)	1.000
	PR	33 (89%)	13 (87%)	20 (91%)	
8 weeks	CR	18 (49%)	10 (67%)	8 (36%)	0.099
	PR	19 (51%)	5 (33%)	14 (64%)	
12 weeks	CR	28 (76%)	14 (93%)	14 (64%)	0.056
	PR	9 (34%)	1 (7%)	8 (36%)	
At last assessment	CR	36 (97%)	14 (93%)	22 (100%)	0.405
	Relapse	1 (3%)	1 (7%)	0	

(*) For the comparison between the IV and SC groups (Fisher’s exact test).

**Table 3A:** Hematologic complications the IV and the SC group.

Parameter/Median (range)	IV (N=18)	SC (N=31)	P value (Wilcoxon test)
**eniBesaB**			
Hemoglobin (g/dl)	8.8 (5.3 to 10.6)	8.2 (4.4 to 10.9)	
Leukocytes (× 10^9^/L)	2.95 (0.80 to 26.20)	2.40 (0.90 to 23.00)	
Neutrophils (×10^9^/l)	0.57(0.07 to 1.10)	0.78 (0.00 to 4.00)	
Platelets (× 10^9^/L)	39 (16 to 98)	33 (3 to 206)	
**Day 10**			
Hemoglobin (g/dl)	8.7 (6.0 to 10.9)	8.5 (5.0 to 15.0)	
Leukocytes (× 10^9^/L)	1.00 (0.32 to 4.28)	1.10 (0.20 to 15.00)	
Neutrophils (×10^9^/l)	0.17 (0.04 to 0.63)	0.15 (0.00 to 1.70)	
Platelets (× 10^9^/L)	40 (4 to 87)	43 (14 to 254)	
**Absolute difference Nadir– Baseline**			
Hemoglobin (g/dl)	0.0 (−2.7 to +3.6)	0.0 (−2.0 to +5.7)	0.324
Leukocytes (× 10^9^/L)	−1.60 (−24.71 to −0.23)	−1.45 (−18.50 to +2.27)	0.459
Neutrophils (×10^9^/l)	−0.30 (−1.01 to +0.33)	−0.48 (−3.02 to +0.90)	0.119
Platelets (×10^9^/L)	−6 (−25 to +7)	−1 (−38 to +239)	0.065

**Table 3B:** Non-hematologic complications the IV and the SC group.

Complication/Number of patients	IV (N=18)	SC (N=31)

Mucositis	12	10

Mucositis G1–2	9	10

Mucositis G3	3	0

Fever and infectious complications overall	14	11

Herpes simplex infection	5	1

Other infections	9	10
≤4 weeks	7	7
>4–10 weeks	2	3

Fever without evidence of infection	4	7

All were G1–2		

Hepatobiliary	0	2

Neurology	0	1

Muscueloskeletal	0	1

Pulmonary (cough)	4	5

Diarrhea	2	3

Nausea	5	9

Vomiting	2	4

Headache	4	6

Musculoskeletal pain	2	3

**Table 3C:** Causes of death in the IV and the SC group.

Cause/Number of patients	IV (N=18)	SC (N=31)
Disease progression and infectious complication	3	0
Disease progression	-	1
Second cancer	0	1
Heart failure and in CR	1	0
Unknown and in CR	1	0

**Table 4:** Prognostic factor analysis of Event Free Survival (EFS).

	P value (Wald Test)	HR	95% CI	
			Lower	Upper

**Univariate analysis**				

Age (>50 versus <=50 years)	0.642	0.731	0.195	2.739

Gender (female versus male)	0.995	0.000	0.000	.

Lymphadenopathy and/or Hepatomegaly (yes versus no)	0.018	4.264	1.286	14.140

Line of treatment (at relapse versus at onset of disease)	0.442	1.676	0.449	6.251

Route (SC versus IV)	0.116	0.386	0.118	1.266
